# The cumulative effects of AI-generated feedback on syntactic development in high school EFL writers: a longitudinal quasi-experimental study

**DOI:** 10.3389/fpsyg.2026.1892877

**Published:** 2026-09-17

**Authors:** Qiong Xiao, Yunhe Sun

**Affiliations:** 1 School of Educational Science, Hunan Normal University, Changsha, Hunan, China; 2 Yali Yanghu Middle School, Changsha, Hunan, China; 3 School of Literature and Digital Communication, Hunan University of Technology and Business, Changsha, Hunan, China

**Keywords:** AI-generated feedback, cognitive load theory, EFL writing, longitudinal quasi-experimental study, skill acquisition theory, syntactic development

## Abstract

**Introduction:**

While AI-generated writing feedback research has flourished, most studies prioritizeholistic quality over syntactic competence and rely on static pre-post comparisons, neglecting dynamic multi-round evolution.

**Methods:**

This 35-week quasi-experiment examined cumulative associations between exposure to supplementary AI-generated feedback components and syntactic development among 69 Chinese high school EFL learners (Group A: *n* = 33 receiving automated AI scoring, official model compositions, and supplementary AI-generated feedback components; Group B: *n* = 36 receiving automated AI scoring and official model compositions). Piecewise regression, cognitive load scales, and qualitative triangulation were used across 10 timed continuation tasks.

**Results:**

The study described non-linear trajectories: an initial adaptation decline followed by faster gains. Among the 10 target error types, only Part-of-Speech errors showed a statistically significant reduction after FDR correction; other error categories exhibited only suggestive trends that did not remain robust after multiple comparison adjustment. Mixed-methods analysis described a hypothesized cognitive load pattern in which extraneous load diminished yet germane processing diverged contingent on learner strategy, with passive offloading coinciding with an illusion of competence. Four revision strategies emerged.

**Discussion:**

The findings are consistent with a conditional pathway within the CLT-SAT framework as an interpretive lens and suggest the potential of a phased Human-in-the-Loop model for contexts where supplementary AI-generated feedback is provided, one that scaffolds autonomy while guarding against passive offloading.

## 1 Introduction

Writing proficiency is widely recognized as a fundamental competency for learners in the 21st century, shaping their ability to communicate effectively, think critically, and succeed academically and professionally (Lahuerta Martínez, 2018). Within English as Foreign Language (EFL) contexts, writing is particularly critical, as it serves as a key indicator of overall language proficiency and a gateway to higher education and career opportunities (Tai and Zhao, 2024). The Gaokao scoring rubric prioritizes grammatical and syntactic accuracy as a key differentiator across all bands (Ministry of Education of the People’s Republic of China, 2020). High scores require few to no errors; any mistake that affects meaning causes immediate downgrading. Accuracy must also coexist with structural richness, as monotonous or error-ridden complex sentences are penalized, making grammar foundational to coherence and overall quality.

Feedback plays an indispensable role in enhancing students’ writing skills, guiding them to identify errors, revise content, and develop linguistic accuracy (Bitchener and Storch, 2016; Bitchener and Ferris, 2012; Hattie and Timperley, 2007). However, in many educational settings, especially in large classes, teachers face significant constraints in providing timely, individualized, and high-quality feedback to each student due to limited time and heavy workloads. This practical dilemma calls for innovative solutions that can supplement traditional teacher feedback and support learners’ writing development at scale.

Recent advances in generative artificial intelligence (GAI) have opened new possibilities for addressing this challenge. Since the launch of ChatGPT in 2022, various GAI tools have gained widespread attention among L2 researchers and practitioners (Ahn et al., 2024; Almanea, 2024). Unlike traditional Automated Writing Evaluation (AWE) systems that primarily detect surface-level errors, GAI can generate tailored, immediate, and detailed feedback that takes learners’ proficiency levels into account, acting as a language consultant, partner, and assessor (Xu and Zhao, 2024). This capability offers a new avenue for improving feedback efficiency in EFL writing instruction.

Existing studies have shown that GAI feedback enhances students’ writing quality, with advantages in speed, personalization, and cost-effectiveness over teacher feedback alone (Alsofyani and Barzanji, 2025; Steiss et al., 2024). However, several critical gaps remain.

First, while most studies have focused on holistic writing quality, specific syntactic competence, such as grammatical accuracy and the reduction of particular error categories, has received far less attention (Polio and Friedman, 2016). This gap is significant because syntactic accuracy is a foundational component of writing proficiency, particularly for beginner and intermediate learners.

Equally importantly, prevailing research relies on static pre–post comparisons, which only capture overall or aggregate gains but fail to capture the dynamic, uneven evolution of writing quality across multiple rounds of GAI feedback or repeated feedback cycles (Zhang et al., 2022; Wang L. et al., 2024; Wang Z. et al., 2024). Whether syntactic knowledge develops linearly, accelerates, or plateaus over repeated feedback cycles remains unexplored. Yet longitudinal designs with multiple measurement points are precisely what is needed to trace such developmental trajectories.

Finally, existing literature has tended to verify the effectiveness of GAI feedback while insufficiently investigating the psychological mechanism that underlies differential effects. Factors such as cognitive load and learning strategies may mediate or moderate how learners benefit from GAI feedback, yet the mechanism has rarely been tested empirically.

To address these gaps, the present study focuses on EFL writing among Chinese senior high school students and conducts a longitudinal quasi-experiment spanning multiple rounds of GAI feedback engagement. This study attempts to: (a) describe the specific cumulative associations between exposure to a multi-round supplementary AI-generated feedback report (comprising AI-generated scores, official model compositions provided by the writing prompt designers, and AI-generated personalized feedback components: error annotations with revision suggestions, alternative expressions, content enrichment, exemplary advanced sentence structures, and exemplary advanced vocabulary) and improving students’ syntactic development, especially accuracy, compared to a control group receiving AI-generated scores and official model compositions; (b) trace the dynamic syntactic developmental trajectories across multiple feedback rounds; and (c) explore the cognitive load patterns that co-occurred with these trajectories in the supplementary feedback condition. It is important to clarify that this study conceptualizes the intervention as a supplementary AI feedback report condition rather than isolated “AI feedback.” Specifically, both groups received automated AI scoring and official reference models; the experimental group exclusively received the AI-generated personalized components embedded within the full report. Consequently, the design tracks cumulative associations linked to the full report package but does not disentangle AI-specific personalization from increased feedback quantity and worked-example exposure.

## 2 Theories and literature review

### 2.1 GAI feedback in EFL writing

For decades, L2 writing research has sought more effective feedback approaches. Traditional methods, from teacher feedback, peer feedback, to AWE, each have documented limitations: teacher feedback is often delayed; peer feedback varies in quality; AWE systems detect surface errors but rarely explain why a structure is incorrect or how to revise it (Lefevre and Cox, 2017; Zhang and Schunn, 2023; Sasaki et al., 2026). GAI feedback, defined as automated, context-sensitive comments generated by large language models, offers a potential solution, providing more timely and effective feedback without corpus training (Steiss et al., 2024).

Existing research on GAI feedback can be divided into three streams. The first emphasizes that collaborative GAI-teacher feedback produces better outcomes than GAI alone. Guo and Wang (2024) compared ChatGPT and teacher feedback, finding that ChatGPT produced significantly more feedback but lacked understanding of specific curricula and student proficiency, highlighting the need for collaboration. Asadi et al. (2025) further demonstrated that hybrid feedback significantly improved IELTS writing performance across all scoring components. However, GAI feedback itself has inherent advantages that warrant independent investigation.

The second stream focuses on specific writing aspects affected by GAI feedback. AI-generated scores correlate well with human ratings in grammar, coherence, and organization (Mizumoto and Eguchi, 2023), and studies have explored applications ranging from grammar correction to essay assessment (Liu et al., 2024; Naismith et al., 2023; Bašić et al., 2023). Yet very few have investigated the development of specific syntactic accuracy. Which error types are most responsive to GAI feedback remains unclear.

The third stream examines psychological mechanisms, yet remains insufficiently theorized. The motivational pathway posits that GAI feedback enhances intrinsic motivation (Teng, 2026; Zare et al., 2025; Song and Song, 2023), but findings are mixed and may plateau over time. The self-regulatory pathway describes how learners convert resources into improvement through cyclical frameworks, yet longitudinal tracking across multiple feedback cycles remains scarce.(Wu et al., 2025; Forsythe and Vargas, 2025) The cognitive pathway suggests GAI reduces extraneous load and reallocates resources (Yao and Fan, 2025), but this remains undertheorized in three respects: GAI introduces novel load sources that have not been systematically examined; whether reduced load operates independently or interacts with motivational mechanisms is unresolved; and optimal load distribution varies by configuration. Collectively, how GAI feedback produces writing gains and the internal mechanisms through which it operates requires further empirical clarification. Emerging evidence from Chinese EFL contexts further substantiates these pathways. Sun et al. (2025) demonstrated that GAI feedback produces value-added effects on college students’ English writing quality, while Mohammed and Khalid (2025) traced how AI-generated feedback simultaneously shapes motivation, emotional stability, and writing development in single-intervention designs. However, neither study tracked syntactic development longitudinally or examined how these mechanisms evolve under sustained, multi-round exposure.

### 2.2 Theoretical framework

#### 2.2.1 Cognitive load theory (CLT) as a Lens for understanding supplementary AI-generated feedback

CLT posits that working memory has limited capacity. Learning is impaired when instructional materials exceed this capacity. CLT distinguishes among three types of cognitive load: intrinsic load (inherent complexity of the material), extraneous load (Load from poor instruction or distracting information), and germane (effort devoted to schema construction and automatization) (Sweller, 1988; Sweller et al., 1998). Critically, extraneous and germane load operate as “communicating vessels,” reducing extraneous load frees resources for germane processing (Paas et al., 2003; Paas et al., 2010).

Empirical evidence supports this mechanism in feedback contexts. Bonilla López et al. (2018) found direct corrective feedback imposed lower cognitive load than metalinguistic feedback, yielding greater long-term grammatical accuracy. Similarly, Wang L. et al. (2024) and Wang Z. et al. (2024) reported that elaborated feedback combining correct and erroneous solutions reduced extraneous and increased germane load, enhancing transfer performance. These findings illustrate the “communicating vessels” principle in action that well-structured feedback reduces extraneous processing demands, thereby liberating cognitive resources for schema-relevant germane load. Among germane load dimensions, authorial core processing, the cognitive effort devoted to preserving narrative voice and macro-level coherence, represents a critical yet underexplored indicator of autonomous execution (Yao and Fan, 2025).

However, these studies neither employed GAI nor tracked load longitudinally, leading to a critical gap, because GAI fundamentally reconfigures writing’s cognitive architecture. Liu et al. (2024) identified novel GAI-specific load sources: prompt management, critical evaluation, and integrative synthesis. Yao and Fan (2025) empirically validated these dimensions, finding critical evaluation the most demanding (M = 4.81). Although visual GAI feedback reduces load and improves coherence (Zou et al., 2025), longitudinal dynamics remain unexplored. Whether initial high extraneous load diminishes with growing GAI literacy, and whether this translates into increased germane load for syntactic learning, is unclear. CLT predicts that reduced extraneous load benefits accumulate progressively as learners internalize feedback and construct automated schemas (Sweller, 2011).

Equally underexplored is the cognitive–affective interplay in GAI-assisted writing. While longitudinal research in traditional writing contexts has demonstrated that writing self-efficacy negatively predicts writing anxiety (*β* = −0.645 for females; *β* = −0.603 for males), thereby indirectly alleviating extraneous cognitive load (Cheng et al., 2026), no study has examined whether this relationship holds in GAI-assisted settings or how iterative GAI feedback might simultaneously reduce extraneous load and enhance self-efficacy over repeated cycles. Methodologically, existing GAI writing research relies predominantly on cross-sectional designs, making it impossible to distinguish stable schema construction from temporary offloading, calling for a shift towards longitudinal empirical paradigms (Wu et al., 2025). As Grinschgl and Neubauer (2022) caution, AI offloading may boost immediate performance while undermining long-term consolidation if germane load is suppressed. This risk is critical for syntactic learning, which requires sustained germane processing for proceduralization (DeKeyser, 2007).

Most critically, no study has examined how cognitive load dynamics relate to specific syntactic errors across multiple GAI feedback rounds. Whether reduced extraneous load facilitates error reduction in particular linguistic domains, or whether some error types persist despite load optimization, remains unknown, a significant gap in understanding how CLT mechanisms translate into tangible writing improvement.

#### 2.2.2 Skill acquisition theory (SAT) as process theory

This study is grounded in Skill Acquisition Theory (SAT; Anderson, 1982; DeKeyser, 2007), which posits that language learning involves a transition from declarative knowledge to proceduralized, and eventually, automated behavior. It describes skill development across three stages: declarative (effortful retrieval), procedural (compiled knowledge requiring less conscious effort), and autonomous (fluent, effortless execution). Informative feedback acts as a catalyst for proceduralization: by providing immediate, targeted input during practice, it enables learners to compile declarative rules into production rules, thereby reducing the cognitive effort required for subsequent execution. Each practice opportunity strengthens the relevant production rule, making it fire more quickly and reliably, while feedback draws attention to target forms and contributes to interlanguage restructuring. Recent empirical work substantiates this mechanism in L2 grammar contexts, where linguistic encoding often consumes significant working memory resources. For instance, DeKeyser (2007) traced the proceduralization of morphosyntactic knowledge through repeated practice and found that sustained, meaningful engagement, not mechanical repetition, drove the transition from controlled to automatic processing. This provides direct empirical backing for the mediating role of practice quality between declarative knowledge and procedural skill. Additionally, the same framework notes that the declarative-to-procedural transition is particularly pronounced when feedback is immediate and task-embedded.

However, these studies neither employed GAI nor tracked proceduralization longitudinally across multiple feedback rounds. This gap is significant because GAI feedback may alter the practice architecture of writing. Emerging research indicates that interacting with GAI introduces novel processing demands, including prompt engineering, critical evaluation of AI output, and integration of AI-generated suggestions (Wu et al., 2025; Yao and Fan, 2025), yet these demands have yet to be examined within a cognitive-load framework. Indeed, Yao and Fan (2025) found that evaluating AI-generated content requires substantial critical thinking, potentially diverting cognitive resources from the germane processing proceduralization requires. A recent narrative review further highlights this methodological gap, identifying longitudinal analyses as a primary direction for future research on GAI and cognitive load in language learning (Li and Alias, 2025). Whether the immediacy and iterability of GAI feedback compress the proceduralization timeline or fragment attention through excessive extraneous load remains largely unexplored. Multi-round GAI feedback designs that track error-type-specific trajectories are therefore urgently needed.

#### 2.2.3 Integrative framework: synthesizing CLT + SAT

Bringing CLT and SAT into dialogue offers a theoretically grounded lens for understanding how multi-round GAI feedback may promote syntactic development. CLT (Sweller, 1988; Paas et al., 2003) explains how focused GAI feedback reduces extraneous load and increases germane load, while SAT (Anderson, 1982; DeKeyser, 2007) specifies how these freed resources drive the declarative-to-procedural transition through repeated practice. Thus, the integrated model posits a potential process in which supplementary AI-generated feedback may optimize working memory capacity via CLT, which then supports the meaningful engagement that SAT requires for knowledge compilation, processes potentially associated with syntactic accuracy.

Despite this theoretical complementarity, these frameworks have rarely been brought into dialogue in GAI feedback contexts. Existing research predominantly employs cross-sectional designs (Wu et al., 2025), leaving it unknown whether the hypothesized synergy manifests in multi-round settings or operates uniformly across different syntactic error types. It is hypothesized that GAI feedback serves as a cognitive scaffold, reducing the extraneous load associated with prompt management and critical evaluation of AI output (Yao and Fan, 2025; Paas and van Merriënboer, 2020), thereby freeing resources for integrative synthesis and authorial core processing. As learners’ GAI literacy increases, extraneous load diminishes, allowing sustained engagement with the deliberate practice that drives knowledge compilation. This progression mirrors the fading procedure central to worked-example research (Renkl, 2014). Notably, while GenAI use boosts learner motivation along a curvilinear trajectory (Teng, 2025), whether cognitive load follows a parallel pattern remains unexamined.

The present study therefore proposes to longitudinally describe the co-occurrence of CLT-predicted load dynamics, SAT-predicted knowledge compilation patterns, and syntactic development during sustained GAI-feedback exposure. A formal test of sequential mediation is beyond the scope of this quasi-experimental design and is reserved for future experimental research.

### 2.3 Research gaps and pathway construction for current study

The integration of CLT and SAT provides a comprehensive framework for understanding how multi-round GAI feedback may promote syntactic development in writing. CLT explains how focused, explanatory GAI feedback optimizes working memory by reducing extraneous load and increasing germane load (Sweller, 1988; Paas et al., 2003), while SAT specifies the trajectory through which these freed resources drive the declarative-to-procedural transition (Anderson, 1982; DeKeyser, 2007).

Despite this complementarity, empirical research has not systematically examined how these mechanisms operate in concert, leaving two critical gaps.

Gap 1: Differential syntactic development under sustained GAI feedback.

Existing studies on GAI writing feedback relies predominantly on static pre–post comparisons, leaving the developmental trajectory of syntactic gains under sustained exposure largely uncharted. Whether cumulative influence of supplementary AI-generated feedback benefits the development of sentence structure in writing and which specific syntactic aspects (such as tense, subject-verb agreement, article usage, etc.) respond to AI-generated feedback awaits empirical specification.

Gap 2: Mediating mechanisms. It is unclear whether supplementary AI-generated feedback is associated with syntactic accuracy alongside cognitive load reduction, proceduralization, or both pathways simultaneously. Without measuring potential mediators such as perceived cognitive load (reduced extraneous load, increased germane load), the field risks claiming effectiveness without understanding how or why GAI feedback works. Specifically, whether perceived cognitive load co-occurs with the relationship between GAI feedback and syntactic development awaits empirical testing.

This study employs an additive quasi-experimental design: both groups receive automated AI scoring, teacher feedback, and official model compositions; the experimental group additionally receives supplementary AI-generated feedback.

Based on the identified gaps in the literature regarding the longitudinal, non-linear impacts of generative artificial intelligence (GAI) on L2 writing, the present study was guided by the following four research questions:

RQ1: How is the provision of the addition of supplementary AI-generated feedback associated with the longitudinal trajectory of high school EFL learners’ overall writing scores?

RQ2: To what extent is this supplementary AI-generated feedback associated with the stage-specific remediation of specific grammatical items, and how do these developmental trajectories diverge across different linguistic features?

RQ3: How is longitudinal engagement with the supplementary AI-generated feedback associated with shifts in learners’ cognitive load across various human-computer interaction and authorial dimensions?

RQ4: How do students perceive and proceduralize supplementary AI-generated feedback, and how do their individual revision strategies contextualize variations in empirical learning outcomes and cognitive burden?

## 3 Method

### 3.1 Research design

This study employed a longitudinal, mixed-methods, quasi-experimental design to investigate the potential cumulative associations between exposure to a supplementary AI-generated feedback and high school students’ syntactic development. A quasi-experimental approach was selected due to the practical constraints of the educational setting and the ethical imperative of allowing voluntary adoption of the supplementary components. Consequently, participants were not randomly assigned to control and experimental conditions; rather, group assignment was determined by the students’ voluntary election to receive the supplementary AI-generated feedback components. To ensure a comprehensive understanding of the intervention’s outcomes, the study utilized a triangulation design, merging quantitative data (performance metrics and survey data) with qualitative insights derived from semi-structured interviews. Detailed intervention components are provided in Section 3.4.

### 3.2 Setting and participants

The initial sample comprised 69 students (32 males, 37 females) drawn from two parallel 12th-grade classes. The research was conducted at a public high school located in a provincial capital of central-southern China, spanning 35 instructional weeks (beginning in the spring semester of 11th grade and concluding in the spring semester of 12th grade). The target population consisted of students following the same English curriculum and preparing for the National College Entrance Examination (commonly known as the Gaokao). They were native Mandarin speakers and had studied English as a foreign language for an average of 10 years.

To control for potential confounding variables related to instructional variation, both classes were taught by the exact same English teacher using identical materials. Participation in the study was strictly voluntary. The writing of all participants was scanned and submitted to the same AI-generated system. Based on their voluntary election to receive supplementary AI-generated feedback, participants were divided into two cohorts:

1. Experimental group (Group A, *n* = 33): Comprising students who voluntarily chose to receive the supplementary AI-generated feedback, which provided individualized error annotations, alternative expressions, content enrichment, exemplary advanced sentence structures, and exemplary advanced vocabulary in addition to the automated scores, official model compositions, and teacher feedback available to all students.
2. Control group (Group B, *n* = 36): Comprising students who chose not to receive the supplementary AI-generated feedback, and thus accessed only the automated AI scoring, official model compositions, and teacher feedback that were uniformly available to both classes.

Prior to the experiment, a 120-min school-based English proficiency diagnostic test (based on the China Standards of English Language Ability) confirmed baseline equivalence between classes. Table 1 presents full demographic and proficiency comparisons. Overall proficiency and initial writing scores did not differ significantly between groups (*p* = 0.487 and *p* = 0.114, respectively), with small effect sizes (Cohen’s d = −0.166 and −0.385). Given the voluntary self-selection design, Inverse Probability of Treatment Weighting (IPTW) was applied using these two continuous covariates (overall proficiency and initial writing score). After weighting, all standardized mean differences were below 0.10 (Table 2), indicating adequate balance.

**Table 1:** Baseline equivalence between groups.

Variable	Group A (experimental group)	Group B (control group)	Test	*p*	Cohen’s d
*n*	33	36	—	—	—
Overall proficiency (Mean)	114.94 (9.82)	117.00 (14.41)	−0.699	0.487	−0.166
Initial writing score (Mean)	16.50 (2.45)	17.49 (2.66)	−1.602	0.114	−0.385

**Table 2:** Covariate balance before and after IPTW adjustment.

Variable	Type	Unadjusted SMD	Adjusted SMD
Overall proficiency (Mean)	Continuous	−0.1671055	−0.017085424
Initial writing score (Mean)	Continuous	−0.3853938	−0.000419072

Informed consent was obtained from both participants and their parents or guardians, in accordance with the ethical guidelines of the Review Board. Beyond procedural consent, safeguards included individual account access with data visible only to the holder, retention limited to June 30, 2026, and parental notification of AI processing for research only with unconditional withdrawal rights. During the intervention, both groups had their writing automatically scored by the GAI system and received the same official model compositions provided by the writing prompt designers, as well as overall evaluations and feedback from the same teacher during class. However, only Group A received detailed, supplementary AI-generated feedback.

### 3.3 Instructional context and materials

To contextualize the students’ writing proficiency, it is important to note their prior and concurrent curricular foundations. During their first and second years of high school, the participants studied the *General High School Textbook: English* (Volumes 1–7, published by Yilin Press and Oxford University Press). In their third year (12th grade), their primary instructional material transitioned to the *Step-by-Step 1st Round Review Book for English*. Furthermore, their curriculum was supplemented with teacher-developed teaching materials explicitly targeted at continuation writing tasks, which included structured learning guides and accompanying instructional courseware rigorously reviewed to ensure uniformity across classes.

### 3.4 Instruments and intervention tool

- *Longitudinal writing tasks*: To quantitatively assess writing progress, performance data were extracted from 10 distinct continuation writing tasks completed over the 35 instructional weeks. Crucially, these 10 writing tasks were not *ad hoc* prompts, but were drawn from real high-stakes examinations: each task was either a previous National College Entrance Examination (Gaokao) writing prompt or a nationally used mock examination prompt. For every task, an official item facility coefficient (ranging from 0.57 to 0.73) published by testing authorities was obtained based on large-scale candidate performance. Thus, the tasks carry established, externally derived difficulty indices, avoiding the uncalibrated nature of arbitrary classroom prompts. Each task was designed to strictly simulate the Gaokao format and consisted of two steps. Within a 40-min time limit, students first read an open-ended English story consisting of approximately 350 words. In the second step, students were required to compose a continuation of the story based on its content and the provided opening sentences of two paragraphs, writing approximately 150 words to create a complete short story (see Appendix A for a sample continuation writing task) (National Education Examinations Authority, 2025, pp. 31–32). The resulting dataset included the objective scores assigned to each writing, as well as detailed statistics regarding the frequency and specific types of errors. These tasks were assessed by the GAI system for both groups.
- *GAI evaluation and feedback system (Intervention Tool)*: Developed in 2024 and implemented for this study in the spring of 2025, this GAI system utilizes natural language processing to automatically tally specific grammatical and structural errors, calculate the total number of errors per submission, and score a student’s writing accordingly (applied to both groups). The scoring system was trained strictly on the established examination rubric for Gaokao writing, meaning that AI scores reflect the degree to which a text conforms to the transparent, official standard rather than to an idiosyncratic algorithmic preference. For the experimental group only, it additionally generates actionable, individualized improvement feedback (see Appendix B for a sample of full GAI evaluation and feedback report). This full AI-generated feedback report comprises the following seven main sections:

1. *Composition score*: Display of the student’s total score (X/25), the highest score, and the class average.
2. *Correction results (Error Annotation)*: Paragraph-by-paragraph annotation of specific errors. Error categories include article, spelling, pronoun, capitalization, noun, tense, subject-verb agreement, clause and part-of-speech, etc. Each error is accompanied by a revision suggestion (original text marked with strikethrough; correct form underlined).
3. *Alternative expressions (Sentence Optimization)*: More idiomatic and concise rewrites for the student’s original sentences, focusing on improving the naturalness of sentence structure and word choice.
4. *Content enhancement*: “Richer Details” adds environmental descriptions, action details, and facial expressions to enhance vividness. “Emotional Deepening” incorporates psychological descriptions and inner monologues. “Thematic Elevation” adds philosophical reflection beyond plot resolution, elevating the narrative to thematic dimensions (e.g., human-animal relationships).
5. *Student exemplary sentences (Advanced Sentence Structures)*: The student’s use of emphatic or complex sentence structures (e.g., cleft sentences) is identified and praised.
6. *Student exemplary sentences (Advanced Vocabulary)*: Highlights of the student’s use of advanced vocabulary, including labels denoting vocabulary difficulty levels, accompanied by affirmative feedback.
7. *Model compositions*: Two or three official model writings provided by the writing prompt designers to demonstrate more complete narratives, richer details, and more sophisticated language use while maintaining the original plot framework.

Both groups received automated AI-generated scoring (total score, class average, and highest score), the same official model compositions provided by the writing prompt designers and overall evaluations and feedback from the same teacher during class after each task. The experimental group received a supplementary AI-generated feedback report additionally comprising individualized error annotations with revision suggestions, alternative expressions, content enrichment, exemplary advanced sentence structures, and exemplary advanced vocabulary. Consequently, the experimental condition is conceptualized as “supplementary AI-generated feedback report exposure” rather than isolated “AI feedback,” and the specific contribution of AI-generated content cannot be disentangled from the additional input dose and worked examples.

- *Human validation scoring*: Human blind scoring validated the automated assessments using three tasks (Tasks 5, 7, 8) completed by students from another parallel class (*n* = 63 per task; total *N* = 189). Two experienced teachers (R1, R2) independently double-scored the writings. Inter-rater reliability was high (ICC(2,1) = 0.850–0.882), supporting the expert average (RM) as a credible reference. AI scores correlated moderately to strongly with RM (Pearson r = 0.682–0.746, all *p* < 0.001), despite systematic lower scores (bias = −1.35 to −2.80) and proportional scale compression (slope = 0.497, 95% CI [0.41, 0.59], intercept = 6.84). Generalizability analyses showed that single-task AI scores had moderate reliability (G = 0.636), while the average of three tasks achieved robust reliability (G = 0.840, φ = 0.783), nearly identical to the human benchmark (G = 0.839, φ = 0.774). Thus, aggregated AI scores provide a reliable metric for tracking relative longitudinal development, though not absolutely equivalent to human ratings without calibration (see Tables 3, 4).
- *Cognitive load scale for non-interactive GAI feedback in EFL writing*: To investigate changes in learners’ cognitive load after receiving AI feedback tools during writing tasks, this study utilized an adapted version of the Cognitive Load Scale for AI-Assisted L2 Writing (CL-AI-L2W; Yao and Fan, 2025), a validated instrument originally developed for assessing cognitive load in AI-assisted L2 writing contexts. Importantly, the scale was administered exclusively to the experimental group because it is explicitly designed to measure cognitive processes specific to interacting with AI-generated feedback reports (e.g., evaluating AI alternative expressions, processing AI error annotations), which the control group did not experience. The adapted scale comprised four subscales: Feedback Reception and Processing (FRP; Items 1–5), Critical Evaluation (CE; Items 6–10), Integrative Synthesis (IS; Items 11–14), and Authorial Core Processing (ACP; Items 15–18). Following the triarchic cognitive load model (Sweller et al., 1998), extraneous cognitive load was operationalized through the FRP and CE subscales (reflecting processing demands evoked by report format and by critical evaluation of AI output), whereas germane load was operationalized through the IS and ACP subscales (reflecting effort directed toward integrating feedback and preserving authorial intent). This operationalization was adapted for the present high-school EFL narrative-continuation context by reconceptualizing the original Prompt Management dimension as FRP and aligning all item wording with post-writing corrective feedback (see Appendix C for the complete item list and wording). Reliability analysis for the present sample (*n* = 33) yielded an excellent overall Cronbach’s alpha of α = 0.943 for the total scale, with subscale alphas ranging from 0.794 to 0.905. Data from the experimental group (*n* = 33) were collected at the pre-test (immediately following the first task) and post-test (after all tasks were completed), spanning the 35-week intervention period.
- *Semi-structured interviews*: Qualitative data were gathered through semi-structured interviews conducted with a subsample of six participants who all came from the experimental cohort on a voluntary basis. The interview protocol (see Appendix D) was designed to elicit deeper insights into the students’ subjective experiences and was structured across four thematic phases: (1) ice-breaking and overall perception, focusing on initial cognitive load and attitude changes; (2) concrete operations and skill internalization, examining how students proceduralized the AI’s feedback despite a lack of explicit grammar explanations; (3) learning outcomes and skill transfer, probing for both conscious and unconscious automatization of error avoidance; and (4) the AI’s scaffolding role, seeking a comprehensive evaluation of its association with teacher reliance and overall writing proficiency.

**Table 3:** Inter-rater reliability (ICC) among human experts and AI system.

Metric	Task 5	Task 7	Task 8
Expert 1 vs. Expert 2	0.882 [0.47, 0.96]	0.879 [0.00, 0.97]	0.850 [0.02, 0.96]
AI vs. Expert 1	0.508 [0.09, 0.74]	0.319 [−0.10, 0.63]	0.516 [0.01, 0.76]
AI vs. Expert 2	0.751 [0.46, 0.87]	0.508 [0.02, 0.75]	0.759 [0.56, 0.86]

ICC (2,1) for absolute agreement, single measurement. Values in brackets represent 95% confidence intervals.

**Table 4:** Criterion validity of AI scores against expert average (RM).

Task	Pearson r [95% CI]	CCC	Bias	SD of Diff.	95% LoA
1	0.729 [0.59, 0.83]	0.626	−1.405	1.836	[−5.00, 2.19]
2	0.682 [0.52, 0.80]	0.403	−2.802	2.355	[−7.42, 1.81]
3	0.746 [0.61, 0.84]	0.639	−1.353	1.691	[−4.67, 1.96]

All Pearson *r* values are significant at *p* < 0.001. Bias, AI – RM. SD of Diff., Standard Deviation of Differences; LoA, Limits of Agreement.

### 3.5 Procedure

The quasi-experiment was conducted over 35 instructional weeks and followed these sequential steps:

1. *Recruitment*: Informed consent was obtained from 12th-grade students and their parents.
2. *Group assignment*: Participants were divided into experimental and control groups based on voluntary self-selection regarding access to the supplementary AI-generated feedback. A baseline English proficiency diagnostic test was administered to confirm equivalence between the two classes.
3. *Intervention introduction*: The intervention began on April 10, 2025. All students were briefed on the study procedures and the AI scoring system. Students in the experimental group were additionally introduced to the supplementary AI-generated feedback features and given the option to use it voluntarily for their mock exams.
4. *Longitudinal testing*: Both groups completed 10 standardized continuation writing tasks simulating the Gaokao over the testing period (concluding on March 10, 2026, excluding the July, August, and February holiday breaks).
5. *Feedback administration*: Following each task, both groups received automated AI-generated scores, official model compositions by the writing prompt designers and overall evaluations and feedback from the same teacher during class after each task. The experimental group was additionally provided with detailed supplementary AI-generated feedback comprising individualized error annotations with revision suggestions, alternative expressions, content enrichment, exemplary advanced sentence structures, and exemplary advanced vocabulary.
6. *Cognitive load measurement*: For the experimental group exclusively, the Cognitive Load Scale was administered at pre-test (immediately following the first writing task) to establish a baseline of their initial reaction to the specific GAI feedback format, and at post-test (following the completion of the 10th mock exam).
7. *Interviews*: Semi-structured interviews were conducted with the six voluntary participants from the experimental group after the intervention period.
8. *Debriefing*: At the semester’s end, participants were debriefed regarding the experimental purpose and offered data withdrawal rights.

### 3.6 Data analysis

A mixed-methods analysis approach was utilized, integrating both quantitative and qualitative techniques. All quantitative analyses, including performance data and questionnaire results, were conducted using the R statistical computing environment via RStudio. The significance level for all statistical tests was set at α = 0.05.

#### 3.6.1 Adjustment for grouping Bias via IPTW

Because a quasi-experimental design with self-selection grouping was utilized, this approach inherently risks grouping bias. To mitigate this self-selection bias, we estimated the probability of receiving the supplementary AI-generated feedback using a logistic regression with two continuous covariates (Overall Proficiency and Initial Writing Score) as predictors. Stabilized inverse probability of treatment weights (IPTW) were computed. Covariate balance was assessed via standardized mean differences (SMD). The standardized mean differences for Overall Proficiency and Initial Writing Score decreased from −0.167 to −0.017 and from −0.385 to −0.0004, respectively, after weighting, indicating adequate balance. This weighted sample was subsequently used in all longitudinal statistical analyses of task scores and grammatical errors to support more robust comparative inferences.

Stabilized IPTWs ranged from 0.70 to 1.67 (M = 1.00, SD = 0.21). One observation was truncated at the 99th percentile (threshold = 1.67). Cook’s D and leverage diagnostics identified five potentially influential cases; however, their presence did not substantially alter the balance results, and the covariates remained well balanced after weighting (all adjusted SMDs < 0.02). The IPTW-based effective sample size for the combined longitudinal sample exceeded 65, while the original group sample sizes remained *n* = 33 (experimental) and *n* = 36 (control), suggesting only minimal precision loss. As a sensitivity check, excluding the five influential cases did not materially change the primary Group × Time interaction for overall writing score; the interaction remained statistically significant and in the same direction.

#### 3.6.2 Analysis of writing performance

To analyze the historical score trends across the 10 writing tasks, two complementary quantitative methods were employed:

1. *Growth curve modeling (GCM)*: Used to assess the global linear trend of variables over time. For the continuous overall English score, a GCM based on the Linear Mixed-Effects Model (LMM) was employed; for grammar errors, which constitute a discrete count variable, a Generalized Linear Mixed Model (GLMM) based on the Poisson distribution was adopted. The “group × time” interaction term (β coefficient) in the model reflects the overall linear slope difference between the two groups across the entire observation period. Error counts were analyzed as raw frequencies because word-count data were unavailable for the control group. Item facility was included as a fixed-effect covariate in the longitudinal mixed models for both the overall score and each error category. In the exploratory Item Response Theory (IRT) analyses, the facility coefficient was entered as a known item parameter (via an offset term) for count-based error models, ensuring that task difficulty was explicitly modeled rather than ignored. Additionally, for the overall score, a graded response model (GRM) was fitted to discretized score categories to explore item discrimination parameters. Because multiple hypothesis tests were conducted across the overall score and 10 distinct error types, the Benjamini–Hochberg procedure was applied to control the false discovery rate (FDR). Adjusted *q*-values are reported, and findings are only considered statistically robust if they achieve *q* < 0.05. Overdispersion in the Poisson GLMMs was evaluated using the Pearson dispersion ratio and DHARMa simulated residuals. If overdispersion was detected, negative binomial mixed models were fitted as a sensitivity analysis. Model fit was compared via AIC and BIC.
2. *Piecewise regression analysis*: Since real cognitive development is often non-linear (e.g., involving adaptation periods, bottlenecks, and insight phases), a single linear slope from the GCM is prone to statistical distortion due to stage-specific fluctuations (for instance, early-stage trial-and-error variability may inflate the global error-rate slope). Piecewise regression was used to detect “breakpoints” where trend mutations occurred in the learning trajectory. By comparing the local slopes before and after the breakpoint (Slope 1 and Slope 2), it describes stage-specific changes in the score trajectory associated with the intervention at specific learning stages. Crucially, to evaluate the validity and stability of these segmented models given the limited 10-point time series, we used bootstrap resampling (1,000 iterations) to estimate the standard errors (SE) and 95% percentile confidence intervals (CI) of the breakpoints. Davies test *p*-values and Akaike Information Criterion (AIC) comparisons against linear models were computed on the original data. Breakpoints were initially identified using the segmented package in R (Version 4.6.1; Muggeo, 2003), with bootstrap medians reported as point estimates.

In addition to overall score analysis, targeted error analysis was conducted. From the aggregate error statistics derived from the 10 tasks, the 10 grammatical items exhibiting the highest overall error rates were selected for detailed analysis. The frequency and remediation rates of these top 10 errors were compared between the two groups to explore whether the supplementary AI-generated feedback supported more rapid or effective correction of persistent grammatical issues compared to the basic AI-scoring, official model compositions and teacher feedback that were uniformly available to both classes.

To verify the reliability of the AI-generated error taxonomy used in the targeted analysis, a stratified random sample of 30 experimental-group writings (10 from early-phase Tasks 1–3, 10 from mid-phase Tasks 4–6, and 10 from late-phase Tasks 7–10; balanced across proficiency levels) was independently coded by two EFL teachers. All AI-flagged error segments were extracted; coders judged whether the AI-assigned category was correct via blind binary judgment (correct / incorrect). In six instances (0.9%) where coders disagreed, a third rater adjudicated. Inter-coder agreement was 99.1% (673/679), and the AI’s overall classification accuracy was 95.3% (647/679).

#### 3.6.3 Analysis of cognitive load

Given the small sample size (*n* = 33) precluding confirmatory factor analysis (CFA) validation of the four-factor structure, the total cognitive load score (18 items, α = 0.943) was analyzed as the primary, most psychometrically defensible outcome measure. Paired-samples t-tests were utilized to assess pre-test to post-test differences, with Cohen’s d reported as the measure of effect size. Furthermore, within-dimension inter-item correlation matrices and corrected item-total correlations (CITC) were calculated to screen for potential redundancy and evaluate item discrimination.

#### 3.6.4 Analysis of qualitative data

A hybrid thematic analysis approach was employed to analyze the qualitative interview data, allowing for deductive categorization based on existing frameworks while remaining inductively open to unexpected nuances in the students’ subjective experiences. This qualitative analysis was later triangulated with the quantitative data. The procedure followed three phases:

1. *Deductive coding (Top-Down)*: An initial codebook was created in direct alignment with the interview phases and theoretical constructs. This included coding for Initial Cognitive Load (e.g., extraneous vs. germane load), Proceduralization (e.g., copying vs. analyzing), Automatization and Transfer (e.g., conscious vs. unconscious error avoidance), and Scaffolding and Autonomy (e.g., shifting teacher reliance).
2. *Inductive coding (Bottom-Up)*: As the transcripts of the six interviewees were reviewed, new codes were generated to capture emergent themes, such as specific emotional responses or unexpected methods of student-AI interaction not covered by the initial theories.
3. *Case profiling*: Because the qualitative sample consisted of exactly six students from the experimental group, each student was treated as an instrumental case study. A qualitative profile was constructed for each participant, summarizing their unique trajectory from initial perception to ultimate skill internalization.

## 4 Results

This section presents the findings of the mixed-methods investigation into the potential impact of the GAI evaluation and feedback system. All quantitative analyses of writing performance and grammatical errors were conducted on the IPTW-weighted sample to account for self-selection bias. The results are organized into four main subsections: overall writing performance trajectories, targeted grammatical error remediation, shifts in cognitive load, and a mixed-methods triangulation of students’ subjective experiences.

### 4.1 Longitudinal writing performance

#### 4.1.1 Growth curve modeling of overall scores

After adjusting for baseline covariates via IPTW and controlling for task facility, the growth curve model (GCM) revealed a significant group × time interaction, *β* = 0.275, SE = 0.071, t = 3.873, *p* < 0.001, FDR *q* = 0.005 (see Table 5). Task facility also showed a significant effect on scores, β = 2.044, SE = 0.330, *t* = 6.186, *p* < 0.001. This indicates that the overall linear growth trajectory of the experimental group was significantly steeper than that of the control group, independent of task difficulty fluctuations.

**Table 5:** Fixed effects estimates for the growth curve model on writing scores.

Predictor	*β*	SE	*t*	*p*	FDR q
Intercept (Ctrl at Time = 0, Facility = 0)	8.286	1.293	6.407	<0.001	—
Group (Exp vs Ctrl at Time = 0)	−2.140	0.548	−3.907	<0.001	—
Time (Ctrl Group Slope)	−0.063	0.052	−1.223	0.222	—
Task Facility (IF)	2.044	0.330	6.186	<0.001	—
Group × Time (Exp Slope Diff)	0.275	0.071	3.873	<0.001	0.005

Dependent variable: Overall AI writing score. Models adjusted for IPTW weights. Time was coded 1–10 and facility was entered uncentered; therefore, the intercept and group coefficient are extrapolated to Time = 0 (and facility = 0 for the intercept) and do not represent baseline scores at the first writing task or the pre-treatment writing test. Baseline equivalence was established separately via the initial writing score (*p* = 0.114, Cohen’s d = −0.385). IPTW weights were stable (range 0.70–1.67; one observation truncated at the 99th percentile). For reference, the model-implied control mean at Task 1 (facility = 0.57) was 15.51. The Task Facility effect is scaled to the minimum–maximum facility in the sample (0.57–0.73). FDR q for Group × Time was computed using the Benjamini–Hochberg procedure across the 11 primary GCM interaction tests (overall score + 10 error types).

#### 4.1.2 Exploratory item response theory (IRT) analysis

An exploratory IRT graded response model (GRM) was fitted to discretized total scores (0–10 = 1, 11–15 = 2, 16–20 = 3, 21–25 = 4). Discrimination parameters (a) were generally adequate for most tasks (range = 0.750–2.502), with the exception of Task 10, which showed a very low discrimination parameter (*a* = 0.138). This suggests possible item misfit or reduced item sensitivity at the end of the 35-week intervention.

#### 4.1.3 Piecewise regression analysis

The piecewise analysis indicated a non-linear trajectory in overall scores. For the experimental group, the bootstrap median breakpoint was at Task 5.16 (SE = 1.12, 95% CI [3.09, 6.34], Davies *p* = 0.017, ΔAIC = −10.95). After this breakpoint, the experimental group’s performance accelerated (Phase 2 slope = 1.225). The control group had a bootstrap median breakpoint at Task 5.59 (SE = 1.34, 95% CI [2.98, 5.98], Davies *p* = 0.273, ΔAIC = −1.75), with a flatter post-breakpoint slope (0.463). Although the uncorrected Phase 2 slope difference was significant (*p* = 0.005), it did not survive FDR correction (*q* = 0.073); therefore, this stage-specific acceleration pattern should be interpreted as exploratory.

Given the limited ten-point time series, the bootstrap CIs for all breakpoints were relatively wide (spanning multiple tasks), indicating that the exact timing of acceleration phases cannot be resolved with high precision. Consequently, the piecewise results should be interpreted as descriptive evidence of potential nonlinear trends rather than as definitive developmental thresholds (see Figure 1).

**Figure 1:**
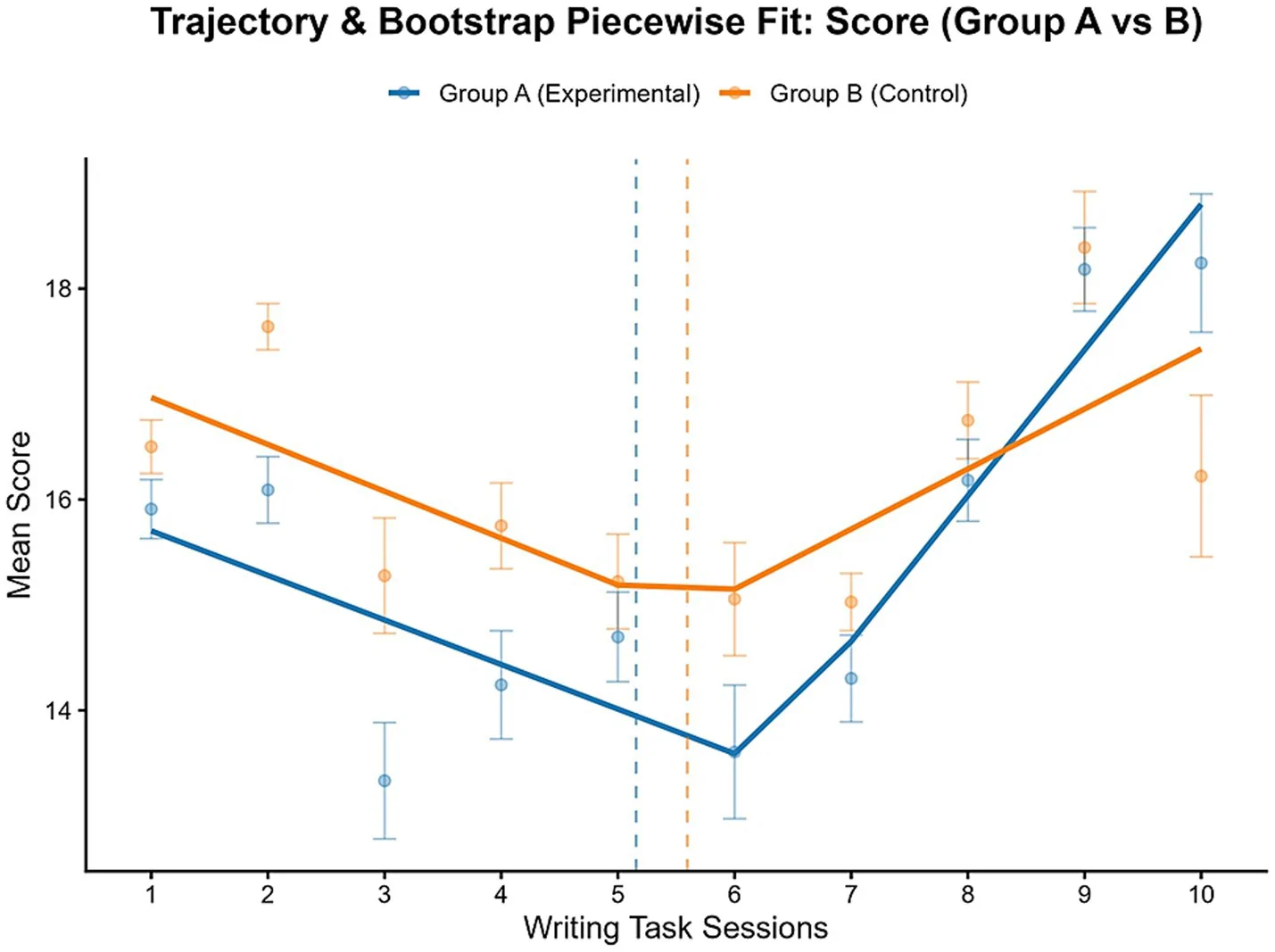
Piecewise regression trajectories for writing scores between groups.

### 4.2 Grammatical error remediation

Detailed analysis was conducted on the 10 grammatical items exhibiting the highest baseline error rates across all participants: Part-of-Speech, Tense, Preposition, Clause, Sentence Component, Pronoun, Singular/Plural, Non-finite verb, Article, and Subject-Verb agreement. A generalized linear mixed model (GLMM) based on the Poisson distribution was used to analyze error counts over time, with task facility and group × time interactions included as predictors.

Task facility was negatively associated with most error types; for example, Part-of-Speech errors showed an incidence rate ratio of 0.588 (95% CI [0.464, 0.745], *p* < 0.001). Even after controlling for task facility, the global linear model revealed a robust group × time interaction for Part-of-Speech errors, β = −0.091, SE = 0.026, z = −3.489, *p* < 0.001, FDR *q* = 0.011, indicating a significantly steeper decline over time in the experimental group. Tense and Clause errors did not reach significance (*p* = 0.068 and *p* = 0.052, respectively). Exploratory IRT models identified pre-FDR group differences for Preposition errors (RPCM *p* = 0.018; binary Rasch *p* = 0.036), but these also failed to remain significant after FDR correction (q = 0.135 and q = 0.198, respectively). Table 6 presents the fixed effect estimates for all 10 error types.

**Table 6:** Fixed effects estimates for the GLMM on all targeted grammatical errors.

Error type	β (Group × Time)	SE	z	*p*	FDR q
Part-of-Speech	−0.091	0.026	−3.489	<0.001	0.011
Tense	−0.032	0.017	−1.823	0.068	0.273
Sentence Component	0.006	0.030	0.200	0.841	0.925
Preposition	−0.022	0.021	−1.050	0.294	0.705
Clause	−0.056	0.029	−1.943	0.052	0.229
Pronoun	0.001	0.030	0.029	0.977	0.983
Singular/Plural	−0.017	0.028	−0.598	0.550	0.878
Non-finite verb	0.013	0.038	0.348	0.728	0.912
Article	−0.012	0.025	−0.490	0.624	0.912
S-V Agreement	0.029	0.053	0.555	0.579	0.878

Overdispersion diagnostics based on Pearson dispersion ratios and DHARMa simulated residuals indicated significant extra-Poisson variation for several error types (e.g., Tense and Singular/Plural, Pearson and DHARMa *p* < 0.001). For the primary outcome (Part-of-Speech), overdispersion was mild and non-significant (Pearson ratio = 1.07, *p* = 0.248; DHARMa *p* = 0.320). Negative binomial mixed models were therefore fitted as a sensitivity analysis. The Group × Time interaction for Part-of-Speech remained significant under the negative binomial specification (β = −0.083, SE = 0.026, z = −3.214, *p* = 0.001, FDR *q* = 0.013 within the sensitivity set of 43 tests). For Tense and Clause, the interactions did not survive FDR correction under either model (Tense NB *p* = 0.027, *q* = 0.178; Clause NB *p* = 0.039, *q* = 0.196). However, negative binomial models for some low-count error types produced boundary estimates or failed to converge; therefore, their AIC/BIC values should not be interpreted as evidence of superior fit. Full diagnostics and model comparisons are provided in Appendix E.

Piecewise regression was applied to explore non-linear stage-specific changes. The validity of breakpoints was assessed with 95% CIs, Davies tests, and AIC comparisons (Table 7; diagnostic details in Table 8). Because several 95% CIs exceeded three tasks and some spanned nearly the full observable 1–10 range, these breakpoints should be interpreted as descriptive indications of trend shifts rather than precise temporal locations.

**Table 7:** Piecewise regression breakpoints and Phase 2 slopes for grammatical errors.

Error type	Experimental breakpoint	Experimental slope 2	Control breakpoint	Control slope 2
Part-of-Speech	7.00	−0.220	4.57	0.074
Tense	4.31	−0.473	6.57	−0.403
Sentence Component	4.38	−0.040	5.72	−0.076
Preposition	6.24	−0.202	8.00	−0.167
Clause	4.56	−0.138	6.00	−0.117
Pronoun	5.00	−0.085	7.00	−0.073
Singular/Plural	8.00	−0.507	8.00	−0.614
Non-finite verb	7.00	−0.243	7.61	−0.275
Article	3.00	−0.113	8.00	−0.355
S-V Agreement	8.69	−0.242	7.00	−0.112

**Table 8:** Validity diagnostics for piecewise regression breakpoints.

Error type	Breakpoint [95% CI]	SE	Davies *p*	ΔAIC (Seg - Lin)	Evidence strength
Part-of-Speech (Exp)	7.00 [6.00, 8.19]	0.940	0.021	−9.251	Strong
Part-of-Speech (Ctrl)	4.57 [4.57, 4.57]	0.348	0.204	−2.152	Weak
Tense (Exp)	4.31 [4.31, 4.31]	0.062	0.016	−11.069	Strong
Tense (Ctrl)	6.57 [4.00, 7.26]	1.173	0.067	−6.465	Weak
Sentence component (Exp)	4.38 [3.00, 8.00]	1.632	0.972	+2.995	Ambiguous/None
Sentence component (Ctrl)	5.72 [4.13, 9.00]	1.170	0.017	−11.465	Moderate
Preposition (Exp)	6.24 [3.00, 8.97]	2.346	0.004	−15.236	Moderate
Preposition (Ctrl)	8.00 [5.00, 8.61]	1.506	0.470	+2.582	Ambiguous/None
Clause (Exp)	4.56 [3.72, 8.69]	1.727	0.258	−0.537	Weak
Clause (Ctrl)	6.00 [2.00, 8.00]	1.494	0.169	−3.436	Weak
Pronoun (Exp)	5.00 [5.00, 5.00]	0.246	NA	NA	Ambiguous/None
Pronoun (Ctrl)	7.00 [2.00, 8.00]	1.793	0.532	+0.316	Ambiguous/None
Singular/Plural (Exp)	8.00 [2.64, 8.99]	2.385	0.225	−2.057	Weak
Singular/Plural (Ctrl)	8.00 [2.00, 9.00]	3.057	0.124	−3.901	Weak
Non-finite verb (Exp)	7.00 [7.00, 7.00]	0.040	0.143	−4.457	Weak
Non-finite verb (Ctrl)	7.61 [5.00, 8.00]	0.956	0.174	−2.973	Weak
Article (Exp)	3.00 [2.00, 9.00]	3.067	0.370	+0.425	Ambiguous/None
Article (Ctrl)	8.00 [8.00, 8.55]	0.273	0.164	−2.828	Weak
S-V Agreement (Exp)	8.69 [8.69, 8.69]	0.207	0.004	−18.650	Strong
S-V Agreement (Ctrl)	7.00 [2.68, 8.00]	0.987	0.021	−11.748	Moderate

Evidence Strength criteria: Strong = Davies *p* < 0.05, CI width ≤ 3 tasks, and ΔAIC < −6; Moderate = Davies *p* < 0.05 but CI width > 3 tasks; Weak = Davies *p* > 0.05 with negative ΔAIC; Ambiguous/None = otherwise. Several bootstrap CIs were degenerate (lower = upper bound), indicating unstable breakpoint estimation; these cases should be interpreted with particular caution.

For Part-of-Speech errors, the experimental group had a bootstrap median breakpoint at Task 7.00 (SE = 0.94, 95% CI [6.00, 8.19]) and a Phase 2 slope of −0.220, whereas the control group had a bootstrap median breakpoint at Task 4.57 (SE = 0.35, 95% CI [4.57, 4.57]) and slope of 0.074. However, the Phase 2 interaction did not survive FDR correction (*p* = 0.018, q = 0.135). For other error types, the experimental group often exhibited earlier breakpoints or steeper Phase 2 slopes (e.g., Tense: 4.31 vs. 6.57; Article: 3.00 vs. 8.00), but none of these differences were statistically significant after FDR correction. Table 7 lists the bootstrap breakpoints and slopes for all error types, and Table 8 provides validity diagnostics. Overall, only the global linear improvement in Part-of-Speech errors remained robust after multiple comparison adjustment.

Complete bootstrap results, including SEs and 95% CIs for all outcome measures, are presented in Appendix F (see Figure 2).

**Figure 2:**
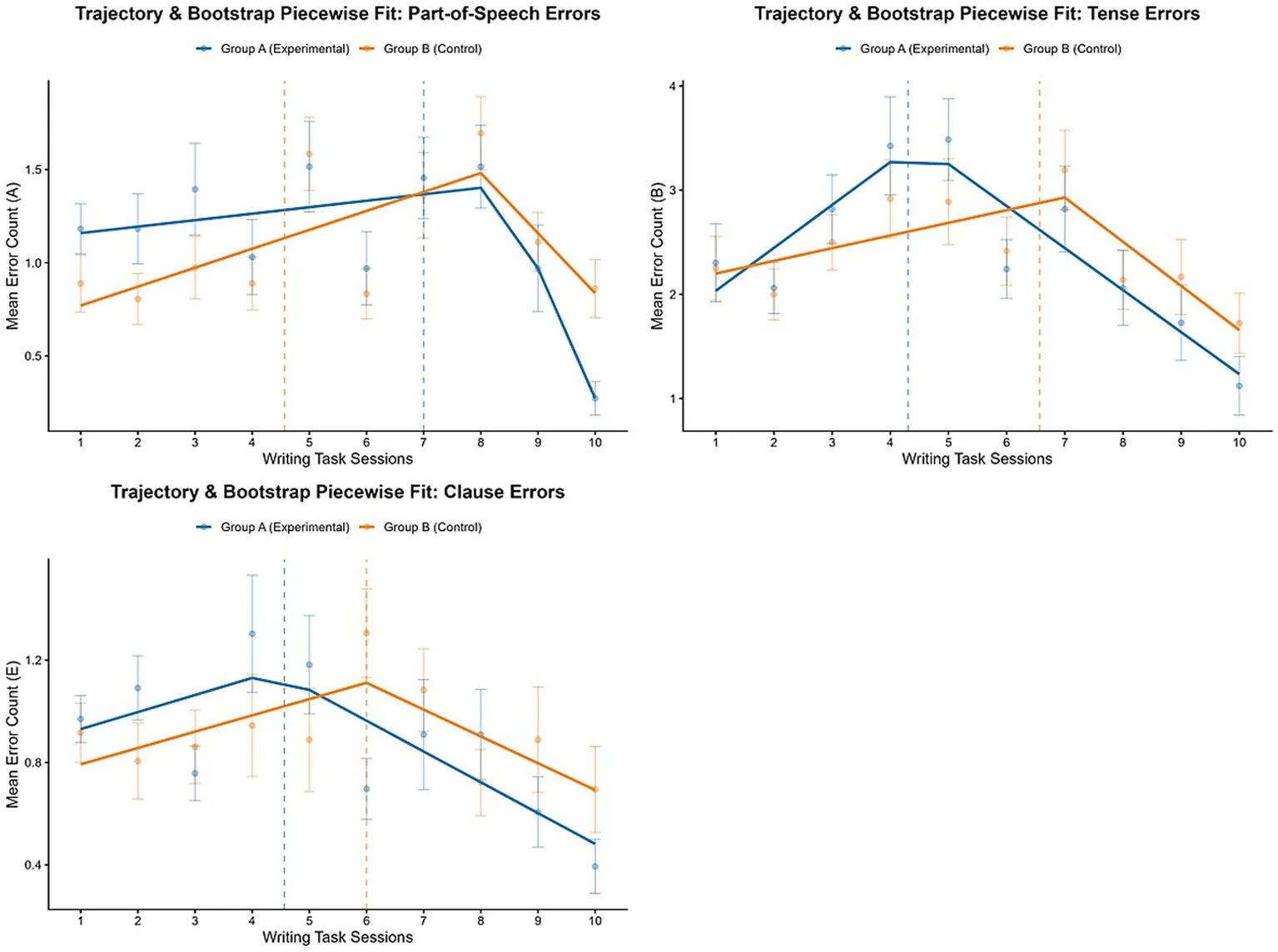
Piecewise regression trajectories for selected grammatical error types (Part-of-Speech, Tense, Clause).

### 4.3 Cognitive load trajectories

Changes in learners’ cognitive load were assessed using the adapted CL-AI-L2W scale administered to the experimental group (*N* = 33) at pre-test and post-test.

The total scale (18 items) demonstrated excellent internal consistency (Cronbach’s α = 0.94). All subscales also showed acceptable to excellent reliability (α range = 0.794–0.905). Corrected item-total correlations exceeded the 0.30 threshold. However, several item pairs showed high inter-item correlations (e.g., Pre_Q1–Pre_Q2, Pre_Q2–Pre_Q3, Pre_Q6–Pre_Q10, Pre_Q15–Pre_Q16, see Table 9), suggesting potential redundancy that should be re-evaluated in larger samples. Because the four-factor structure could not be validated via CFA due to the small sample, subscale results are reported as exploratory.

**Table 9:** Internal consistency and item analysis for the adapted CL-AI-L2W scale (*N* = 33).

Scale/dimension	Items	Cronbach’s α	CITC Range	High inter-item correlation pairs (r > 0.80)
Total scale	18	0.943	0.416–0.792	—
Dimension A (FRP)	5	0.905	0.77	Pre_Q1–Pre_Q2 (*r* = 0.821), Pre_Q2–Pre_Q3 (*r* = 0.890)
Dimension B (CE)	5	0.872	0.70	Pre_Q6–Pre_Q10 (*r* = 0.870)
Dimension C (IS)	4	0.857	0.71	—
Dimension D (ACP)	4	0.794	0.61	Pre_Q15–Pre_Q16 (*r* = 0.842)

The total cognitive load score decreased significantly from pre-test (M = 80.79, SD = 19.24) to post-test (M = 69.09, SD = 15.53), t (32) = 5.53, *p* < 0.001, Cohen’s d = 0.96, indicating a large effect. All four dimensions also showed significant decreases (see Table 10), with Dimension B (Critical Evaluation) exhibiting the largest subscale reduction (d = 0.83). Figure 3 presents the individual pre-test to post-test trajectories for each cognitive load dimension, illustrating that the group-level reductions were accompanied by substantial inter-individual variability. While these reductions are statistically robust within the experimental cohort, the absence of a control group precludes causal attribution solely to the GAI intervention.

**Table 10:** Descriptive statistics and paired-samples *t*-test results for cognitive load (*N* = 33).

Scale/dimension	Pre-test M (SD)	Post-test M (SD)	Mean difference	t(32)	*p*	Cohen’s d
Total (18 items)	80.79 (19.24)	69.09 (15.53)	−11.70	5.53	<0.001	0.96
Dimension A	21.33 (6.72)	18.64 (5.97)	−2.69	3.35	0.002	0.58
Dimension B	23.52 (6.58)	19.12 (5.95)	−4.40	4.74	<0.001	0.83
Dimension C	19.24 (4.68)	16.79 (4.99)	−2.45	3.38	0.002	0.59
Dimension D	16.70 (4.16)	14.55 (4.15)	−2.15	3.29	0.003	0.57

**Figure 3:**
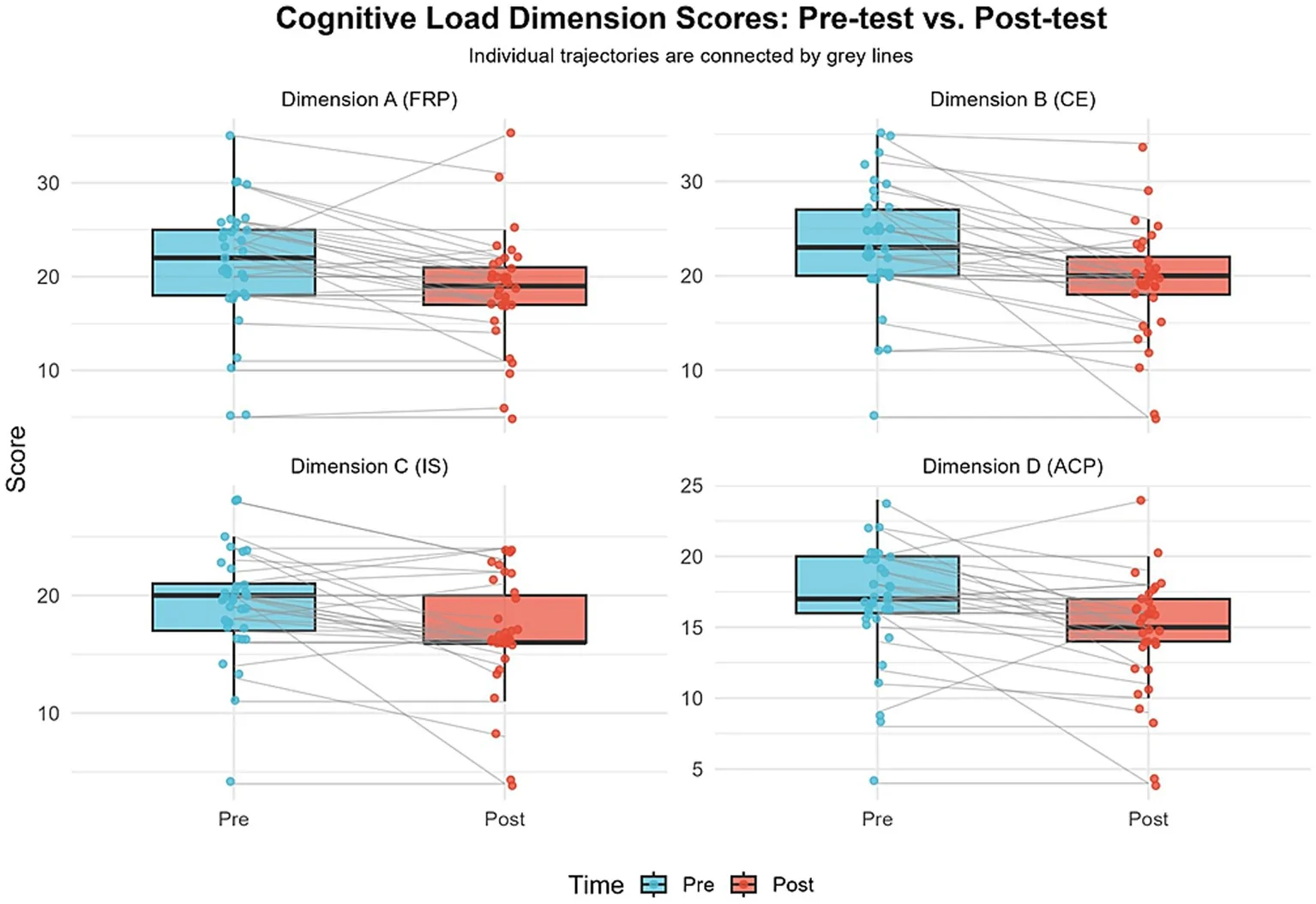
Cognitive load dimension scores: Pre-test to post-test with individual trajectories.

### 4.4 Mixed-methods triangulation: cognitive processing profiles and strategy variation

A hybrid thematic analysis (Fereday and Muir-Cochrane, 2006) was conducted on interview transcripts from six purposively sampled Grade 12 students (Student 1–Student 6). These data were triangulated with item-level cognitive load responses and longitudinal writing task scores to illustrate how subjective processing strategies contextualize quantitative trajectories; the qualitative findings are illustrative rather than statistically representative.

Table 11 presents the integrated matrix of six case study participants and reveals marked heterogeneity masked by group-level statistics; for instance, total cognitive load decreased for three students (Δ = −2.44 to −0.39) but increased for two (Δ = +0.67, +0.50).

**Table 11:** Mixed-methods integration matrix of case study participants.

Student	Quant: Longitudinal Slope (Tasks 1–10)	Quant: Cognitive Load Δ (Post - Pre)	Qual: Dominant Revision Strategy	Qual: Perceived Transfer
Student 1	+0.31 (Flat to Steep Late Positive)	−2.44 (Significant Decrease)	Analyzes underlying grammar rules	High conscious avoidance
Student 2	+0.49 (Stagnant/Fluctuating to Positive)	+0.67 (Slight Increase)	Combative synthesis; untangling semantic errors	Low transfer, repeats errors
Student 3	+0.29 (Flat/Dip then Sharp Positive)	−1.17 (Significant Decrease)	Skeptical engagement, rigid adherence	Retains heavy authorial reliance on human logic
Student 4	+0.39 (Smooth Steady Positive)	−1.83 (Significant Decrease)	Passive reception of complex sentences	Experiences cognitive fatigue, high illusion of competence
Student 5	+0.51 (Dip then Sharp Positive)	−0.39 (Minimal Decrease)	Deep processing, structural comparison	Enhanced self-regulation, prompts strategic teacher dialogue
Student 6	+0.36 (Fluctuating to Positive)	+0.50 (Slight Increase)	Selective application (grammar focus only)	Discards AI structural edits to maintain personal authorial flow

Cognitive Load Δ = Post-test minus Pre-test average scores. Negative values indicate a desirable decrease in cognitive load. Positive values indicate an increase. Longitudinal slopes were calculated using ordinary least squares regression across all 10 tasks.

#### 4.4.1 Temporal alignment of cognitive load

By cross-referencing Phase 1 qualitative interviews with the cognitive load scale data, a precise temporal alignment emerges between subjective feelings and empirical burden.

The Baseline: Retrospective accounts of initial exposure to supplementary AI-generated feedback strongly correlated with pre-test scale variances. Student 1 recalled intense initial resistance: “To be honest, my head exploded when I first opened that report… The whole report was extremely long, and I didn’t want to read it through at all.” Triangulation quantitatively aligns with this: S1’s initial Feedback Reception and Processing (FRP) score was critically high (averaging 6.2/7 on Q1-Q5). Conversely, Student 2 recalled the initial feedback as clear; correspondingly, their baseline FRP score was a remarkably low 2.4/7.

The Delta: Comparing students’ reflections to the post-pre mathematical difference (Δ) reveals the trajectory of adaptation versus friction. Over 35 weeks, S1 noted they eventually found the tool “really fragrant” (highly useful), matching a monumental drop in their cognitive load (Δ = −2.44).

In stark contrast, S2 and S6 experienced long-term increases in cognitive load, but for distinctly different reasons. S2 gradually realized the AI was twisting their original semantic intent: “I found it sometimes a bit ‘self-righteous’, it would change my sentence based on its own understanding, but the result was not what I originally meant at all.” Quantitatively, this friction manifested as a severe burden; S2’s Critical Evaluation score rose sharply from 4.0 to 5.8, and their Integrative Synthesis (the effort required to blend AI feedback without losing one’s voice) increased substantially from 4.5 to a near-maximum 6.25. This suggests S2 was not merely “copying” the AI, but rather fighting a strenuous cognitive battle to untangle the AI’s misinterpretations, resulting in a net load increase (+0.67). Similarly, S6 experienced a load increase (+0.50) due to authorial protection. Because S6 actively discarded complex structural edits to maintain their voice (“I will complain to the teacher that the AI didn’t understand my train of thought, and then ask the teacher to help me sort out how to structure the writing more smoothly”), their Integrative Synthesis and Critical Evaluation burdens increased markedly. This illustrates that selectively filtering AI suggestions may demand a significantly higher cognitive toll than passive acceptance.

#### 4.4.2 Correlating behavior with longitudinal trajectories

Integrating qualitative data with the empirical outcomes of the 10 sequential writing tasks describes the relationship between revision strategies, longitudinal growth, and specific error remediation.

Strategy vs. Slope: Analysis of the longitudinal task trends (Tasks 1–10) is consistent with the interpretation that deep cognitive processing temporarily coincided with performance before sharp gains. Students who reported actively analyzing errors or engaging in deep structural comparison (e.g., S1, S5) exhibited volatile or flat Phase 1 slopes followed by a steep Phase 2 acceleration. This trajectory is consistent with a theoretical interpretation of germane cognitive load associated with restructuring linguistic schemas.

Conversely, students employing highly divergent strategies, such as S4’s “passive reception” versus S2’s “combative synthesis,” exhibited distinct slopes. S4’s reliance on the supplementary AI-generated suggestions generated what may be termed an “illusion of competence” – smooth performance gains unaccompanied by deeper understanding. S4 stated: “Facing the ‘excellent sentence analysis’ and ‘sentence structure upgrades’ given by the AI, I find it hard to understand and replicate myself… I basically just memorize and apply them directly.” This rote memorization coincided with immediate, smooth score benefits (steadily reaching 20) but appeared to bypass the productive struggle typically associated with genuine skill acquisition, as reflected in offloading the synthesis burden (which dropped from 6.25 to 3.5). This inference is based on the convergence of qualitative self-report and quantitative cognitive load patterns; direct metacognitive measures in future research would provide stronger confirmation. S2, battling the AI’s semantic assumptions, experienced a heavily stagnant middle phase (scoring 15, 17, 16, 15, 15) before finally adapting to secure a 20.

Error-Specific Triangulation: Students’ qualitative reports of their own progress were consistent with the broader piecewise regression results. Student 1 explicitly noted that “some low-level grammar and part-of-speech errors are now rarely committed.” This offers a qualitative illustration consistent with the massive delayed effect identified in the piecewise model where Part-of-Speech errors experienced a steep acceleration in remediation during Phase 2 (experimental Phase 2 slope = −0.220).

#### 4.4.3 Shifting autonomy: the “cold” algorithm and the human touch

Finally, the matrix describes how students adapted their reliance on human teachers as a coping mechanism for the AI’s limitations. S3 noted the AI was emotionally disconnected: “It feels a bit ‘cold’. It’s like an emotionless machine that only dead-picks grammar and spelling, but it doesn’t seem to understand the coherence and rationality of the article.” S5 responded to this limitation by redirecting their cognitive resources toward high-level exam strategy, utilizing the AI’s advanced structural outputs to prompt teacher dialogues: “I will take the ‘personalized model writing’ given by the AI to discuss with the teacher, asking ‘Do you think if I’m in the exam, is it safer to use my original phrasing, or is the AI’s complex inverted sentence more likely to get a high score?’” As S4 summarized regarding advanced phrasing: “I feel I still need the teacher’s ‘human touch’ guidance.” Ultimately, while exposure to supplementary AI-generated feedback coincided with remediation of surface-level errors, students remained highly reliant on the human touch to evaluate narrative coherence and mitigate the cognitive friction introduced by the algorithm.

## 5 Discussion

The present study employed a 35-week longitudinal, mixed-methods design to investigate the cognitive mechanisms through which supplementary AI-generated feedback is associated with EFL learners’ syntactic development. By adjusting for self-selection bias via IPTW and triangulating piecewise regression data with in-depth cognitive load scale responses and qualitative interviews, this study seeks to describe the complex, non-linear interplay between extraneous load reduction, germane processing, and proceduralization of GAI-Feedback-assisted writing contexts.

### 5.1 Non-linear trajectories of syntactic development

Addressing RQ1, the Growth Curve Modeling (GCM) indicated that the experimental group exhibited a significantly steeper overall growth trajectory than the control group. However, the piecewise regression analysis highlighted that this overarching linear trend did not fully capture the underlying non-linear developmental reality.

#### 5.1.1 Empirical growth patterns

The experimental group’s trajectory exhibited a breakpoint at Task 5.16, with a substantially steeper post-breakpoint slope (Slope 2 = 1.225), indicating delayed acceleration. This delayed acceleration aligns with skill acquisition theories, which suggest that integrating a novel, dense technological tool requires an initial phase of schema restructuring. As evidenced by the qualitative data, students initially experienced high extraneous cognitive load (e.g., Student 1 reporting their “head exploded” when navigating the dense AI reports). It appears that only after learners proceduralized the feedback format, filtering noise and focusing on actionable corrections, resources were reallocated from tool deciphering to linguistic processing, potentially creating conditions for post-breakpoint gains.

#### 5.1.2 Dialogue with existing literature

These findings extend Sun et al.’s (2025) university-based evidence to high-school sentence-level skills. While Sun et al. (2025) found value-added effects on holistic writing quality, the present study describes that exposure to detailed supplementary AI-generated feedback components is associated with significant cumulative benefits in overall writing scores and selective syntactic features, particularly part-of-speech errors. The divergence from Mohammed and Khalid (2025), who reported pretest–posttest gains in GAI-mediated versus non-GAI conditions, highlights a critical boundary condition: GAI literacy maturation functions as a rate-limiting factor. Students’ initial difficulty in parsing dense GAI feedback gave way to more strategic utilization around Iteration 5, corroborating Yao and Fan’s (2025) finding that critical evaluation imposes substantial initial cognitive demands while demonstrating that such load diminishes as learners develop autonomous strategies. This progression responds to Wu et al.’s (2025) calls for longitudinal tracking of GAI-specific processing demands, a dynamic process that single-intervention designs cannot capture.

### 5.2 Stage-specific remediation and the boundaries of GAI efficacy

Regarding RQ2, the study found that supplementary AI-generated feedback is not associated with the uniform remediation of all specific grammatical items, suggesting potential boundaries to the tool’s current pedagogical efficacy.

#### 5.2.1 Accelerated vs. delayed remediation

Piecewise regression revealed distinct temporal patterns for different grammatical items (automated classification validated at 95.3% accuracy; see Method). Descriptively, certain discrete syntactic features such as Tense and Clause showed earlier experimental-group breakpoints (Tasks 4.31 and 4.56, respectively), suggesting a possible tendency toward earlier remediation of these error categories. However, the between-group differences in these piecewise slopes did not survive FDR correction and should therefore be interpreted as exploratory. Descriptively, the experimental group showed a later breakpoint for Preposition errors than for Tense and Clause, but the group × phase interaction did not remain significant after FDR correction. Part-of-Speech errors showed a delayed breakpoint at Task 7.00 following a significant linear decrease (GCM: β = −0.091, FDR q = 0.011), indicating that although initial remediation was slow, the eventual within-group acceleration was dramatic. Because prepositions and part-of-speech derivations in English rarely follow transparent form-function mappings, their mastery often requires extensive exposure to contextualized usage and deep vocabulary knowledge. The delayed breakpoints indicate that learners likely required prolonged, iterative exposure to the AI’s “Alternative Expressions” before pattern recognition and proceduralization occurred.

#### 5.2.2 The limitations of sentence-level AI

Crucially, the GLMM results indicated no significant group × time interaction indicating a GAI advantage over traditional instruction alone for Pronouns, Articles, Singular/Plural nouns, Non-finite verbs, or Subject-Verb Agreement. Articles, Plurals, SVA, and Non-finite verbs likely reflect deep L1 interference (Mandarin lacks articles, plural morphology, and verb conjugations), which sentence-level scaffolding cannot readily overcome. Pronoun errors further require macro-level anaphora resolution across paragraphs, exceeding the tool’s sentence-level scope.

#### 5.2.3 Dialogue with existing literature

This finding extends Steiss et al. (2024) and Guo and Wang (2024), who concluded that GAI comprehensively addresses language features. More precisely, the present study suggests that uptake may follow error-specific timelines determined by cognitive processing demands. From a cognitive load perspective, reductions in extraneous processing burden could, in principle, free cognitive resources for the proceduralization under certain conditions (Sweller et al., 1998; Yao and Fan, 2025). Consistent with this theoretical possibility, the descriptive patterns reported in Section 5.2.1 tentatively suggest earlier breakpoints for some discrete syntactic features (e.g., Tense, Clause) compared to L1-entrenched or discourse-dependent features, although these were exploratory.

### 5.3 Cognitive load reconfiguration and learner strategy

#### 5.3.1 Overall reduction and divergent pathways

In response to RQ3, the total cognitive load score decreased significantly from pre-test to post-test, t(32) = 5.53, *p* < 0.001, Cohen’s d = 0.96. Yet individual trajectories diverged sharply. The mixed-methods triangulation described a critical hidden cognitive pattern in which while extraneous load declined, germane processing, including Integrative Synthesis and Authorial Core Processing, followed divergent trajectories across learners.

Students 1, 3, and 5 showed decreased load (Δ = −2.44, −1.17, and −0.39), while S4 also showed a large decrease (Δ = −1.83) but under a passive-offloading pattern, as discussed below. S1 recalled that their “head exploded” when first navigating the dense report, yet eventually found the tool highly useful, matching the quantitative drop. S3 perceived the algorithm as an “emotionless machine that only dead-picks grammar,” but nonetheless achieved a marked decrease, suggesting that affective distance does not preclude extraneous load reduction. S5 redirected effort toward teacher consultation, using AI-generated models to prompt strategic dialogue. Conversely, Students 2 and 6 experienced increased burden (Δ = +0.67 and +0.50). S2 resisted the algorithm’s “self-righteous” semantic distortions, while S6 defended narrative voice against structural edits that ignored their “train of thought.” This pattern suggests that GAI-mediated feedback may elevate surface-level performance while coinciding with reduced cognitive engagement necessary for genuine writing skill acquisition.

#### 5.3.2 Passive offloading and the illusion of competence

A contrasting pattern was observed in Student 4, who showed a large load reduction (Δ = −1.83) but through passive offloading rather than deepened processing: S4 admitted to directly applying GAI complex syntax without parsing underlying mechanics. S4’s strategy appears consistent with a stalled declarative-to-procedural transition. By rote-memorizing and directly applying AI syntax without parsing mechanics, S4 bypassed the germane load, the productive struggle necessary for schema building. Rather than compiling production rules, S4 remained capable of reproducing complex forms without autonomous execution. This passive offloading coincided with improved text products but limited evidence of genuine skill acquisition. The case illustrates that equivalent empirical score improvements were accompanied by vastly different levels of cognitive engagement, exposing an illusion of competence invisible to score-only metrics.

#### 5.3.3 Dialogue with cognitive load theory

These findings are consistent with the integrative model hypothesized in Section 2.2.3 as an interpretive lens. Rather than the predicted uniform sequential mediation, the data describe a conditional pattern that extraneous load diminishes, yet germane load may rise or fall depending on learner agency. This suggests a potential refinement to the prevailing CLT assumption that reducing extraneous load automatically frees resources for germane processing (Paas and van Merriënboer, 2020). The present study describes a pattern in which extraneous load reduction coincided with a window for potential skill development, and whether this window translates into genuine skill acquisition depends on the learner’s protection of germane load. This corroborates Grinschgl and Neubauer’s (2022) caution that cognitive offloading may diminish deeper engagement and substitute algorithmic output for genuine schema construction.

### 5.4 Student perceptions and revision strategies

Building on the cognitive load reconfiguration identified in RQ3, RQ4 examines how individual processing strategies contextualize these divergent trajectories.

#### 5.4.1 Four distinct strategies

Qualitative analysis identified four distinct revision strategies. (a) Deep analytical processing (S1, S5): learners dissected AI feedback, comparing grammatical structures. Quantitatively, this showed initial score volatility but strong final gains and overall load reduction of varying magnitude (S1: Δ = −2.44; S5: Δ = −0.39). (b) Combative synthesis (S2): learners actively resisted semantic misinterpretations, resulting in elevated cognitive load (Δ = +0.67) and mid-phase stagnation but eventual robust transfer. (c) Passive offloading (S4): learners rote-memorized and directly applied AI suggestions without parsing mechanics, yielding smooth immediate gains and a large overall cognitive load reduction (Δ = −1.83), but this reduction reflected passive offloading rather than deepened processing. (d) Selective filtering (S6): learners applied only grammar fixes while discarding complex structural edits to preserve narrative flow, balancing moderate growth with increased burden (Δ = +0.50). These findings illustrate that equivalent empirical score improvements were accompanied by vastly different levels of genuine skill transfer, suggesting that the effectiveness of supplementary AI-generated feedback may vary with the learner’s metacognitive approach.

#### 5.4.2 Unintended adaptations and the human touch

This Human Touch pattern was not manipulated but emerged qualitatively. First, S4 exhibited surface-level performance without autonomous execution by treating AI feedback as a declarative repository to be reproduced rather than a scaffold to be processed. Score-only metrics cannot detect this risk, underscoring the necessity of complementary process measures to distinguish genuine schema construction from algorithmic dependency. Second, selective filtering was observed in S6, who discarded complex structural edits because the GAI “didn’t understand my train of thought”, explicitly prioritizing authorial protection. While this represents a concerning pattern, it was reported by a single case and should not be generalized. The perception of GAI as emotionally detached nonetheless redirected students toward teachers for macro-level coaching. S5 utilized AI outputs to prompt strategic teacher dialogue, while S4 sought the teacher’s “human touch.” As this GAI assumed sentence-level correction, students elevated teachers to logic coaches and authorial validators, a Human-in-the-Loop dynamic observed across multiple cases.

#### 5.4.3 Dialogue with existing literature

These four strategies suggest that existing conceptualizations of feedback processing may need extension to account for GAI-specific modes such as combative resistance and selective filtering. Within the CLT-SAT integrative framework, deep processing is associated with critical evaluation into germane load, whereas offloading coincides with diminished germane load, consistent with DeKeyser’s (2007) contention that mechanical repetition does not accompany proceduralization. The S6 case further aligns with broader human-computer interaction research on automation bias, wherein users alter behavior to satisfy algorithmic expectations, though this connection should be treated as illustrative rather than confirmatory given the single-case basis. Such compliance raises construct validity concerns that scores may increasingly reflect machine-alignment rather than genuine proficiency, while suppressing the linguistic risk-taking necessary for Zone of Proximal Development (ZPD) advancement. Conversely, the perceived “coldness” of algorithmic feedback (S3) redirected learners toward teachers for discourse-level validation, suggesting that supplementary AI-generated feedback elevates, rather than replaces, the human pedagogical role. Collectively, these findings suggest that supplementary AI-generated feedback effectiveness is not determined by tool design alone, but also appears to depend on the metacognitive stance learners adopt toward that tool. GAI feedback may not render human teachers obsolete. Instead, it could elevate their pedagogical role. As the AI took over the role of automated ‘grammar police’, students redirected their teacher interactions toward high-level logic, emotional narrative coherence, and exam strategy.

### 5.5 Pedagogical implications

The findings suggest a potential, context-bound three-phase structure for integrating supplementary AI feedback reports in high school EFL writing instruction. These boundaries are heuristic and approximate (e.g., overall breakpoint at Task 5.16; earlier for Tense/Clause; later for Part-of-Speech/Preposition).

Phase 1 (approximately Iterations 1–5): Initial cognitive friction appeared prevalent. Independent navigation focused on comprehension rather than scores may help, with attention to rote-memorization risks.

Phase 2 (approximately Iterations 6–8): As students adapted, needs appeared to shift toward narrative coherence. Requiring learners to justify AI-suggestion acceptance warrants testing as a check against passive offloading.

Phase 3 (approximately Iterations 8–10): Selective engagement and self-directed revision emerged among active monitors. Metacognitive prompts (e.g., “What did you learn?”) warrant testing to distinguish acquisition from compliance.

In this sample, supplementary AI-generated feedback appeared to shift the teacher’s role toward discourse-level coaching. Whether this reconfiguration generalizes depends on institutional context and tool design; longitudinal evaluation remains essential, as early dips reflected adaptation rather than lack of learning.

Practical implications. The GAI Evaluation and Feedback System offers clear efficiency gains over human raters, particularly for handwritten texts in time-constrained Gaokao preparation. With typical class sizes, the GAI system correction frees teachers to focus on macro-level logic. However, the documented AI-human score gap warrants calibration so students do not treat algorithmic scores as exam proxies. Under high-stakes pressure, passive offloading risk rises; requiring learners to justify AI suggestions before adoption may mitigate this. Finally, the 48% opt-out rate (36/69) signals that stakeholder orientation, clarifying that AI feedback augments rather than replaces teachers—is essential for scaled implementation.

### 5.6 Limitations and future directions

Several limitations bound the interpretive scope of these findings, and each simultaneously identifies a productive avenue for extending this line of inquiry.

First, the quasi-experimental design cannot isolate the specific effect of AI-generated personalization from the confounding influence of increased feedback dosage and worked-example exposure, as detailed in the Method section. The experimental condition is best understood as supplementary AI-generated feedback report exposure rather than a pure AI-versus-no-AI comparison. Future research could employ dismantling designs varying dosage and worked-example density to isolate active ingredients and inform adaptive feedback design.

Second, due to ethical constraints regarding tool adoption, voluntary self-selection may have introduced unmeasured confounders (e.g., motivation, technology acceptance) that the quasi-experimental design cannot fully rule out, despite overall proficiency and initial writing scores being balanced via IPTW. A randomized controlled trial would provide stronger causal evidence in the future research.

Third, the sample size was modest, which limited the power to detect small effects and precluded CFA of the cognitive load scale. Future large-scale validation of the CL-AI-L2W factorial structure could yield a short-form scale suitable for classroom monitoring.

Fourth, the cognitive load data were collected only from the experimental group, so no between-group comparison could be made. This precludes direct between-group comparison of cognitive load and, critically, prevents formal statistical mediation of the hypothesized CLT to SAT pathway. Nevertheless, the within-group trajectories offer a descriptive benchmark for how learners cognitively negotiate sustained supplementary AI-generated feedback. Future research should administer parallel cognitive-load instruments to both conditions, enabling longitudinal mediation modeling to test whether reductions in extraneous cognitive load and increases in germane engagement mediate the relationship between supplementary AI-generated feedback and writing gains. Process-tracing methods, including stimulated recall and revision trace analysis, could additionally provide objective behavioral data to validate cognitive offloading patterns.

Fifth, the intervention lasted 35 weeks, but the long-term durability of the observed gains remains unknown. Moreover, the Gaokao preparation context may limit generalizability to less exam-driven settings. Future research could include follow-up assessments to examine whether the benefits of supplementary AI-generated feedback persist after the intervention ends.

Sixth, the study focused on one AI tool and one task type. The generalizability of findings to other GAI systems and writing genres requires further investigation.

Additionally, error counts were analyzed as raw frequencies because word-count data were unavailable for the control group; consequently, these trajectories should be treated as exploratory, and future studies should employ text-length offsets to permit rate-based GLMMs.

## 6 Conclusion

This 35-week quasi-experimental study provides evidence that supplementary AI-generated feedback components are associated with faster overall writing-score growth and selective EFL syntactic improvement, most robustly in part-of-speech errors, through non-linear, stage-specific trajectories. The findings describe a cognitive load pattern: reduced extraneous load is associated with a conditional window for proceduralization, with genuine acquisition potentially related to sustained germane engagement. This pattern is consistent with the CLT-SAT framework as an interpretive lens, suggesting boundary conditions for the efficacy of supplementary AI-generated feedback. Pedagogically, these exploratory findings point to a potential phased support structure: scaffolding GAI literacy early, attending to germane load mid-phase, and guarding against passive offloading late. Qualitative observations suggest the potential for repositioning teachers as discourse coaches rather than replacements, an implication consistent with the Human-in-the-Loop framework but drawn from student-teacher co-reliance patterns that await systematic validation. These quasi-experimental findings await validation through randomized designs and formal mediation analysis.

## Data Availability

The raw data supporting the conclusions of this article will be made available by the authors, without undue reservation.
