# Supplementary material for: The cumulative effects of AI-generated feedback on syntactic development in high school EFL writers: a longitudinal quasi-experimental study
Source: Front Psychol. 2026 Sep 17;17:1892877. doi: 10.3389/fpsyg.2026.1892877 (PMC13627100; doi:10.3389/fpsyg.2026.1892877)
Supplement: Supplementary file 1 [file Supplementary_file_1.docx]

**Appendix A: Sample Continuation Writing Task**

**Section 2 (25 points)**

**Read the passage below. Based on the given content and the opening sentence of each paragraph, write two paragraphs to complete the story.**

I met Gunter on a cold, wet and unforgettable evening in September. I had planned to fly Vienna and take a bus to Prague for a conference. Due to a big storm, my flight had been delayed by an hour and a half. I touched down in Vienna just 30 minutes before the departure of the last bus to Prague. The moment I got off the plane, I ran like crazy through the airport building and jumped into the first taxi on the rank without a second thought.

That was when I met Gunter. I told him where I was going, but he said he hadn’t heard of the bus station. I thought my pronunciation was the problem, so I explained again more slowly, but he still looked confused. When I was about to give up, Gunter fished out his little phone and rang up a friend. After a heated discussion that lasted for what seemed like a century, Gunter put his phone down and started the car.

Finally, with just two minutes to spare we rolled into the bus station. Thankfully, there was a long queue still waiting to board the bus. Gunter parked the taxi behind the bus, turned around, and looked at me with a big smile on his face. “We made it,” he said.

Just then I realised that I had zero cash in my wallet. I flashed him an apologetic smile as I pulled out my Portuguese bankcard. He tried it several times, but the card machine just did not play along. A feeling of helplessness washed over me as I saw the bus queue thinning out.

At this moment, Gunter pointed towards the waiting hall of the bus station. There at the entrance, was a cash machine. I jumped out of the car, made a mad run for the machine, and popped my card in, only to read the message: “Out of order. Sorry.”

**Note:**

(1) The number of words in your continuation should be about 150.

(2) Please write your answers on the answer sheet in the following format.

Paragraph 1: I ran back to Gunter and told him the bad news. _______________________________

Paragraph 2: Four days later, when I was back in Vienna, I called Gunter as promised. ___________

**Appendix B: Sample** **GAI Evaluation and Feedback Report**

**Appendix C: Sample Cognitive Load Scale for Non-Interactive GAI Feedback in EFL Writing**

**Instructions: The following questions ask about the amount of mental effort you invested in different activities while using the GAI feedback and applying it to your writing. Please rate your mental effort for each activity on the scale provided. There are no right or wrong answers. Please answer honestly based on your real experience.**

Scale:

1 = Very, very low mental effort

2 = Very low

3 = Low

4 = Moderate

5 = High

6 = Very high

7 = Very, very high mental effort

Part I: Feedback Reception and Processing

1. Understand the grammar error markings, word and sentence modification suggestions, scores and word count statistics, and correction symbols (e.g., strikethrough, underline, error type labels) in the GAI correction results.

2. Compare the “Alternative Expressions” sentences with your own original text, and identify specific word replacements or sentence structure adjustments.

3. Understand the explanation of revision reasons in “Content Enhancement” (e.g., adding descriptive details, deepening emotions, elevating the ending), such as “adding environmental description to enrich details.”

4. Review the “Exemplary Student Sentences” and “Composition Samples” and identify the advanced vocabulary or sentence structures within them.

5. Integrate the feedback information (e.g., correction results, content enhancement, exemplary sentences) in the current round of GAI feedback, and form improvement strategies for your grammar errors and sentence structures.

Part II: Critical Evaluation

6. Judge whether the information provided by the GAI feedback was factually accurate.

7. Evaluate whether the suggestions in the GAI feedback were relevant to the topic of your writing.

8. Assess the tone and style of the GAI feedback to see if it was appropriate for the content of your writing.

9. Decide which parts of the GAI feedback to use and which parts to ignore or delete.

10. Check the content of the GAI feedback contained any inappropriate or illogical elements.

Part III: Integrative Synthesis

11. Paraphrase or rewrite the sentences generated by the GAI, expressing them in your own words.

12. Blend the GAI feedback smoothly with your own original writing.

13. Ensure that your personal authorial voice was not lost when using GAI content.

14. Connect your own ideas logically with the ideas or text provided by the GAI.

Part IV: Authorial Core Processing

15. Decide on the main topic or writing angle for your writing.

16. Create a logical structure or outline for the entire writing.

17. Find the right vocabulary to express your specific ideas precisely.

18. Construct grammatically correct English sentences.

**Appendix D: Sample Interview Questions Protocol**

**Phase 1: Ice-breaking & Overall Perception (Tracking Attitudes and Initial Load)**

1. Can you recall how you felt when you first read comments and feedback the comments and corrective feedback provided by the AI on your essay? Was it easy to understand? After using it for this period of time, how have your feelings about the AI’s comments and feedback changed compared to the initial stage?

2. When the AI gives you feedback and suggestions each time, do you think the amount of feedback is too much, too little, or just right? What kind of feedback makes you understand immediately, and what kind of feedback makes you feel “not quite able to understand”?

**Phase 2: Concrete Operations & Skill Internalization (Exploring Practice and Proceduralization)**

3. When you receive the AI feedback, what steps do you usually take to revise your essay? Do you directly copy the AI’s corrected sentences, or do you first think about why the error occurred?

4. The AI’s corrective feedback does not explain the grammar or vocabulary usage. Instead, it presents the errors in your expression alongside the corrected versions. Does this kind of feedback help you revise and write your essays? Why or why not? Please give an example.

5. In terms of part-of-speech usage, clause usage, pronoun usage, singular/plural, non-subject-verb, article usage, preposition usage, sentence component, tense usage, and subject-verb agreement usage, which aspects do you find the AI feedback has been most helpful for you? Which aspects have been less helpful? Can you provide examples?

**Phase 3: Learning Outcomes & Skill Transfer (Validating Automatization and Competence Development)**

6. After using AI correction feedback have you noticed any changes in your essay writing process? For example, do you consciously try to avoid some some errors that you often made before while writing? What kinds of errors? Do you unconsciously try to avoid some some errors that you often made before while writing? What kinds of errors?

7. Besides helping you correct errors, do you think the AI feedback has enabled you to learn some new, even advanced vocabulary or sentence structures that can be used in the next writing task? Can you give an example from your recent essay?

**Phase 4: Scaffolding Role & Comprehensive Evaluation (Open-ended Reflection)**

8. With the availability of AI feedback, has the frequency of your questions to your English teacher about writing changed? Has the content of your questions changed? If so, how are the questions you ask your teacher now different from before?

9. Overall, compared to the time without AI feedback, do you find revising your essays easier or more difficult now? Why? Since you started using AI feedback, what specific improvements do you perceive in your English writing proficiency?

10. If you were to suggest one improvement for this AI corrective feedback tool, what aspect would you most like it to do better?

**Appendix E Bootstrap Piecewise Regression Summary for Overall Score and Error Types**

| **Variable (Group)** | **Breakpoint (Median)** | **SE** | **95% CI Lower** | **95% CI Upper** | **Davies p** | **ΔAIC (Seg – Lin)** | **Slope1** | **Slope2** |
| --- | --- | --- | --- | --- | --- | --- | --- | --- |
| Score (Experimental) | 5.16 | 1.12 | 3.09 | 6.34 | .017 | –10.95 | –0.924 | 1.225 |
| Score (Control) | 5.59 | 1.34 | 2.98 | 5.98 | .273 | –1.75 | –0.404 | 0.463 |
| Part-of-Speech (Experimental) | 7.00 | 0.94 | 6.00 | 8.19 | .021 | –9.25 | 0.020 | –0.220 |
| Part-of-Speech(Control) | 4.57 | 0.35 | 4.57 | 4.57 | .204 | –2.15 | –0.083 | 0.074 |
| Tense (Experimental) | 4.31 | 0.06 | 4.31 | 4.31 | .016 | –11.07 | 0.758 | –0.473 |
| Tense (Control) | 6.57 | 1.17 | 4.00 | 7.26 | .067 | –6.46 | 0.199 | –0.403 |
| Sentence Component (Experimental) | 4.38 | 1.63 | 3.00 | 8.00 | .972 | 2.99 | 0.207 | –0.040 |
| Sentence Component (Control) | 5.72 | 1.17 | 4.13 | 9.00 | .017 | –11.47 | 0.069 | –0.076 |
| Preposition (Experimental) | 6.24 | 2.35 | 3.00 | 8.97 | .004 | –15.24 | 0.008 | –0.202 |
| Preposition (Control) | 8.00 | 1.51 | 5.00 | 8.61 | .470 | 2.58 | 0.043 | –0.167 |
| Clause (Experimental) | 4.56 | 1.73 | 3.72 | 8.69 | .258 | –0.54 | 0.106 | –0.138 |
| Clause (Control) | 6.00 | 1.49 | 2.00 | 8.00 | .169 | –3.44 | 0.046 | –0.117 |
| Pronoun (Experimental) | 5.00 | 0.25 | 5.00 | 5.00 | NA | NA | 0.065 | –0.085 |
| Pronoun (Control) | 7.00 | 1.79 | 2.00 | 8.00 | .532 | 0.32 | 0.040 | –0.073 |
| Singular/Plural (Experimental) | 8.00 | 2.39 | 2.64 | 8.99 | .225 | –2.06 | 0.053 | –0.507 |
| Singular/Plural (Control) | 8.00 | 3.06 | 2.00 | 9.00 | .124 | –3.90 | 0.115 | –0.614 |
| Non-finite Verb (Experimental) | 7.00 | 0.04 | 7.00 | 7.00 | .143 | –4.46 | 0.033 | –0.243 |
| Non-finite Verb (Control) | 7.61 | 0.96 | 5.00 | 8.00 | .174 | –2.97 | –0.011 | –0.275 |
| Article (Experimental) | 3.00 | 3.07 | 2.00 | 9.00 | .370 | 0.42 | 0.125 | –0.113 |
| Article (Control) | 8.00 | 0.27 | 8.00 | 8.55 | .164 | –2.83 | 0.041 | –0.355 |
| S-V Agreement (Experimental) | 8.69 | 0.21 | 8.69 | 8.69 | .004 | –18.65 | 0.056 | –0.242 |
| S-V Agreement (Control) | 7.00 | 0.99 | 2.68 | 8.00 | .021 | –11.75 | 0.065 | –0.112 |

**Appendix F Overdispersion Statistics and Model Comparison**

| **Error Type** | **Overdispersion Ratio²** | **Overdispersion p** | **DHARMa Dispersion Ratio** | **DHARMa p** | **AIC Poisson** | **AIC NB** | **ΔAIC (Poisson – NB)** | **BIC Poisson** | **BIC NB** | **ΔBIC** |
| --- | --- | --- | --- | --- | --- | --- | --- | --- | --- | --- |
| Part-of-Speech | 1.0686 | .248 | 1.0614 | .320 | 1884.33 | –62545.36³ | 64429.69³ | 1911.56 | –62513.60³ | 64425.16³ |
| Tense | 1.5010 | < .001 | 1.4885 | < .001 | 2763.37 | 2480.46 | 282.91 | 2790.59 | 2512.22 | 278.37 |
| Sentence Component | 1.0153 | .784 | 1.0058 | .920 | 1652.90 | –41245.26³ | 42898.16³ | 1680.12 | –41213.50³ | 42893.63³ |
| Preposition | 1.0714 | .216 | 1.0681 | .256 | 2242.68 | –50480.70³ | 52723.39³ | 2269.90 | –50448.95³ | 52718.85³ |
| Clause | 1.0328 | .568 | 1.0248 | .656 | 1705.55 | –3057.55³ | 4763.10³ | 1732.77 | –3025.79³ | 4758.56³ |
| Pronoun | 1.0442 | .456 | 1.0371 | .504 | 1645.83 | –13150.76³ | 14796.59³ | 1673.05 | –13119.01³ | 14792.06³ |
| Singular/Plural | 1.3240 | < .001 | 1.3237 | < .001 | 1897.71 | 1767.02 | 130.69 | 1924.93 | 1798.77 | 126.15 |
| Non-finite Verb | 0.9951 | .920 | 0.9939 | .928 | 1317.26 | –36593.00³ | 37910.26³ | 1344.48 | –36561.24³ | 37905.72³ |
| Article | 1.0968 | .088 | 1.0826 | .160 | 1930.53 | –1908.73³ | 3839.26³ | 1957.75 | –1876.98³ | 3834.73³ |
| S-V Agreement | 1.1694 | .064 | 1.1863 | .040 | 920.93 | 916.37 | 4.56 | 948.15 | 948.13 | 0.02 |
